# The PAPHIO study protocol: a randomised controlled trial with a 2 x 2 crossover design of physical activity adherence, psychological health and immunological outcomes in breast cancer survivors

**DOI:** 10.1186/s12889-020-08827-x

**Published:** 2020-05-15

**Authors:** Supa Pudkasam, Meron Pitcher, Melanie Fisher, Anne O’Connor, Nanthaphan Chinlumprasert, Lily Stojanovska, Remco Polman, Vasso Apostolopoulos

**Affiliations:** 1 Institute for Health and Sport, Victoria University, Melbourne, VIC Australia; 2 Bernadette de Lourdes School of Nursing Science, Assumption University, Bangkok, Thailand; 3 Breast Cancer Service, Western Health, Melbourne, VIC Australia; 4 IPC Health Altona Meadows, Melbourne, VIC Australia; 5 Department of Food, Nutrition and Health, College of Food and Agriculture, United Arab Emirates University, Al Ain, UAE; 6 School of Exercise and Nutrition Sciences, Faculty of Health, Queensland University of Technology, Brisbane, Qld Australia

**Keywords:** Breast cancer survivor, Self-directed physical activity, Physical activity adherence, Motivational interviewing, Pedometer, Quality of life, Psychological health, And immunological biomarker

## Abstract

**Background:**

The PAPHIO study; a randomized controlled trial with 2X2 crossover design will implement a self-directed physical activity program in which participants will engage in self-monitoring and receive motivational interviewing to enhance physical activity adherence. The study aims to determine the effects of 24 weeks self-directed activity combined with motivational interviewing (MI) on (i) psychological health, (ii) quality of life (QoL) and (iii) immune function in female breast cancer survivors.

**Methods:**

The study will recruit 64 female breast cancer survivors within 3 years of diagnosis and at least 6 months post primary treatments at Western Health Sunshine Hospital, Melbourne, Australia. They will be randomly allocated to immediate intervention (IIG group) or delayed intervention groups (DIG group) in a 1:1 ratio. All participants will be given a wearable device (Fitbit Alta HR) and undertake self-directed physical activity for 24 weeks and will receive MI for 12 weeks (IIG; during week 0 to week 12 and DIG; during week 13 to week 24). Participants’ daily step count and the changes of immune cell functionality will be assessed at the beginning (week 1: T1), week 12 (T2) and week 24 (T3) of the program. Physical activity adherence will be assessed at T2 and T3. Participants will also complete four questionnaires assessing exercise self-regulation (BREQ2), exercise barrier and task self-efficacy, mental health (DASS-21) and QoL (FACT-B) at three time points (T1 to T3). Linear-mixed models will be used to assess the relationship between physical activity volume by step counting and mental health (DASS-21), QoL (FACT-B), immune biomarkers, self-regulation (BREQ2) and self-efficacy at T1, T2 and T3;between 2 groups.

**Discussion:**

We expect this physical activity intervention to be acceptable and beneficial to the participants in terms of psychological and immunological well-being with the potential outcomes to be implemented more widely at relatively low cost to these or other patient populations.

**Trial registration:**

Australian New Zealand Clinical trials Registry- ACTRN12619001271190. Prospectively registered on 13 September 2019.

## Background

Breast cancer is a common malignant disease leading to physical and psychological distress in females worldwide [1, 2]. Approximately 2.1 million women suffered from this disease and 626,000 deceased across the world in 2018 [3]. . The occurrence rate of breast cancer has dramatically increased in 22 out of 39 countries from 2008 to 2012, whereas the global death rate has gradually dropped [4]. The global trend of breast cancer increases awareness in the availability of breast cancer care program [4].

Breast cancer survivors may have mental distress mainly due to long-term treatments [5].Currently, survivorship programs, especially physical activity programs have emphasised strategies to enhance psychosocial well-being [6, 7]. Psychological interventions including emotional ventilation, adjustment skill training and self-efficacy promotion techniques have been applied in improving their mental wellness over the decades [6, 8].

Physical activity, especially moderate intensity aerobic exercise for female breast cancer survivors, have been noted in a number of studies to be beneficial to breast cancer outcomes, decreasing the mortality rate by > 30% and reducing the recurrence rate [9]. As a result of physical activity, there is a reduction in the total body fat as well as a number of inflammatory and immunological biomarkers which could contribute to better outcomes in breast cancer survivors [10, 11]. Moderate aerobic exercise and combination of aerobic and resistant training in breast cancer women ranging between 15 and 24 weeks could possibly activate immune cells such as NK cell cytotoxic activity and lymphocytes [12, 13]. Furthermore, a 12-weeks aerobic exercise training at home in breast cancer survivors reduces the level of epithelial neutrophil activating protein and pro-inflammatory cytokines [14].

Approximately one third of patients with cancer reduce their physical activity after diagnosis and almost 70% of them will not reach the exercise recommendation for cancer patients [15].. 30% Of breast cancer survivors with early phase (stage 0 to 3) breast cancer have reported physical activity cessation during 12 months follow-up after participation in a 6 months RCT [16]. The cessation may be associated with the resumption of their previous domestic tasks and work [16]. Physical activity barriers in older breast cancer survivors are related to physical tiredness and lack of time management skills [17]. In addition, some breast cancer survivors have low confidence in the benefits of physical activity in minimizing adverse effects of breast cancer and treatment [18]. The challenge of recruiting patients with advanced stage of cancer is to deal with their cancer-related fatigue in particular the side effect of treatments [15].

### Physical activity and motivation for activity adherence in breast cancer survivors

Adoption and adherence to physical activity programs amongst cancer survivors are challenging due to their physical and mental vulnerability [19]. As such, in this study we developed an intervention which maximizes feasibility, sustainability and generalisability. This study will prescribe self-directed physical activity to breast cancer survivors. Many of self-directed techniques for physical activity adherence in participants with advanced stage of heterogeneous cancers have been used in research studies, such as partially advised and home-based program, exercise class teaching and peer support walking group programs [20]. Many of the programs have achieved high percentages of exercise adherence, reducing fatigue and improving QoL [20].

An important factor to adopt and adhere to a physical activity program is an individual’s motivation. To this end, a number of behavioural change strategies have been reported which enhance motivation and adherence [21]. Two of these strategies are Motivational Interviewing (MI) and self-monitoring. MI is a conversation technique used by a professional during consultation when making health behaviour changes [22], and has been effectively used to bring about behavioural change in health promotion programs for the general population. This includes changes in eating behaviour, alcohol cessation and adoption of an active life style [23]. More specifically, face-to-face and phone based MI has been implemented successfully to enhance self-efficacy and reduce resistance against physical activity in breast cancer survivors [24, 25].. On the other hand, self-monitoring,one of the important concepts in self-regulation theory, is an auditing mechanism of individual performance in relation to an individual’s cognitions, beliefs and emotions [26]. Some digital devices have the ability to promote physical activity through self-monitoring concept [27]. For example, step counting gadgets including pedometers, can effectively monitor physical activity in terms of daily steps. Such devices have been utilised for promotion of physical activity in clinical trials [28] especially in breast cancer patients and survivors [29, 30]. Step counters help in monitoring individual’s physical activity behaviour and can result in increased motivation and ultimately adherence to long-term physical activity behaviour [31].

To understand participants’ motivation to self-directed physical activity, self-determination theory (SDT) has been effectively used to enhance insight in physical activity behaviour and motivation in breast cancer survivors [32]. According to SDT motivation to engage in behaviour lies on a continuum ranging from extrinsic (controlled by external factors) to intrinsic (individual interest and preference). The theory predicts that intrinsic-motivation might enhance confidence in task accomplishment (competence), independent action (autonomy) and the feeling of connection to others (relatedness) [32]. Our study will assess how self-regulation and motivational interviewing might change participants motivational orientation over the intervention period using the behavioural regulations in exercise questionnaire (BREQ2 [33];).

Self-efficacy, an important concept in social cognitive theory, has been shown to be a predictor for physical activity behaviour and adherence in breast cancer patient [34]. In this project two types of efficacy beliefs will be examined. First, we will explore exercise barriers self-efficacy, which will examine the participants’ confidence to overcome or deal with barriers to their exercise participation. Secondly, this project will examine task self-efficacy beliefs In particular, it will examine the self-efficacy beliefs of the participants to engage in exercise behavior [34]. To measure this, the project will use the nine-item barriers self-efficacy [34] and four-item task self-efficacy questionnaire [34].

### Study goals and objectives

The study has an overall aim to determine the effects of 24 weeks self-directed activity combined with motivational interviewing on (i) psychological health (depression, anxiety, stress), (ii) quality of life (QoL; physical, social/family, emotional and functional) and (iii) immune function in female breast cancer survivors. In addition, we will explore the dose-response relationship between exercise volume (step count) and the outcome measures.

### Study design

**Fig. 1:**
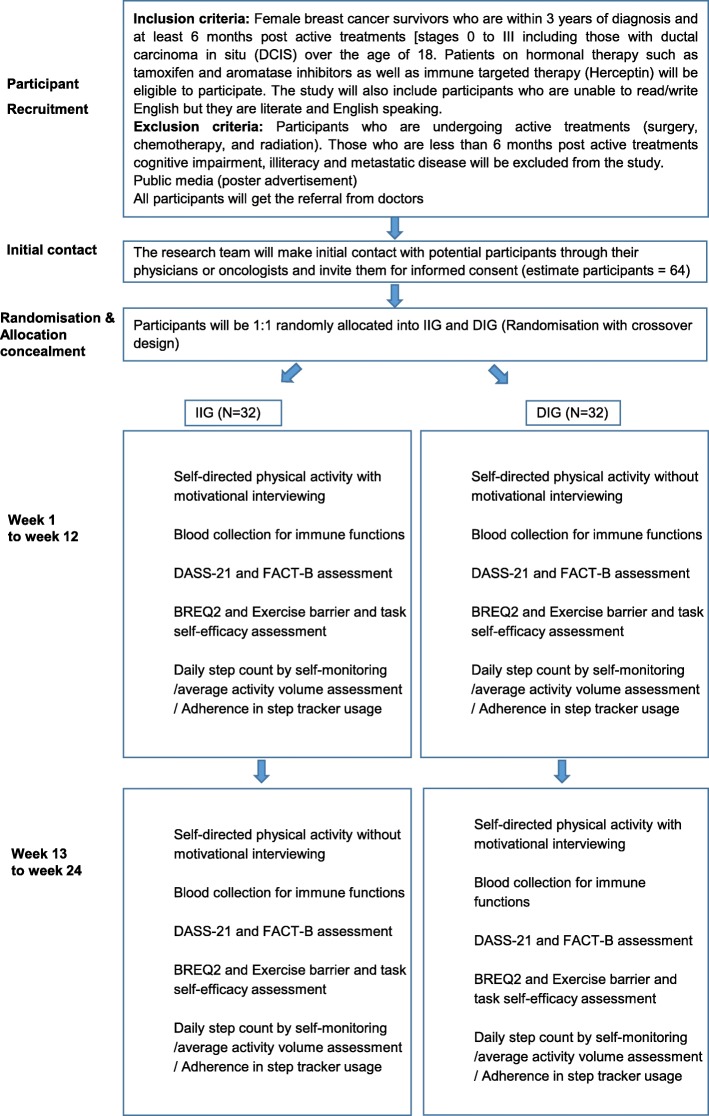
Study protocol through 24 weeks trial

This study is a randomised crossover trial; a single site research project conducted at Brest Cancer Service Clinic, Western Health (Sunshine Hospital) Australia. Potential participants will be prescribed with a Fitbit Alta HR tracker for a 24-week-self-directed activity and will receive motivational interviewing for 12 weeks (Immediate intervention group; during week 0 to week 12 and Delayed intervention group; during week 13 to week 24). The study protocol through 24 weeks is illustrated in Fig. 1.

#### Inclusion criteria

Female breast cancer survivors 18 years and older who are within 3 years of diagnosis and at least 6 months post active treatment; operation, chemotherapy and/or radiotherapy [stages 0 to 3 including those with ductal carcinoma in situ (DCIS)], will be recruited. Survivors on hormonal therapy such as, tamoxifen and aromatase inhibitors as well as immune targeted therapy (Herceptin) will be eligible to participate because these hormonal and cell surface blockages do not have direct effects on immune cells and adiposity-related biomarkers in breast cancer. The study will also include participants who are unable to read/ write English but are literate and English speaking. For participants with non-English literacy, translators will be used to obtain informed consent and the study will apply the questionnaires that have been validated in other languages such as Vietnamese, Greek, Chinese and Macedonian. This study plans to recruit participants from Breast cancer care service of Sunshine Hospital, Western Health, Melbourne, Australia.

#### Exclusion criteria

Participants who are undergoing active treatments (surgery, chemotherapy and radiation) as well as those less than 6 months post active treatment. Likewise, those individuals with cognitive impairment and unable complete the questionnaires will be excluded. Participants with known metastatic disease will also be excluded from the study. The participant will be withdrawn from the study if they develop cancer recurrence or metastases during the study period.

### Recruitment

The study will recruit participants who are breast cancer survivors within 3 years of diagnosis and at least 6 months post primary treatments. Eligible participants will be randomly allocated to immediate intervention or delayed intervention groups in a 1:1 ratio after completion of informed consent. The participants will be recruited from Breast cancer care service, Western Health Sunshine campus in Melbourne. Recruitment strategies will include public media (poster advertising or flyer) and individual contact introduced by their physician [35]. All participants have to be approved by their physician for engagement in the physical activity program of this research and the screening process will be conducted before obtaining consent.

The study will commence at Western Health Sunshine campus following ethical approval from Melbourne Health Human Research Ethics Committee (MH HREC) and Western Health research governance authorization [36]. Based on our power calculation we aim to recruit 64 participants.

### Randomisation

The study design is a two-armed RCT with a crossover design. Sequence generation will be conducted by simple randomisation following patient informed consent. The participants will be randomly allocated to either IIG or DIG in 1:1 ratio by computerised number generator. Allocation concealment by enclosing the allocation in sealed envelopes will be used to prevent selection bias [37]. The allocation concealment will be conducted by a third party to prevent researchers from affecting group assignment. The participants in both groups will be prescribed a self-directed physical activity for 24 weeks. The IIG will receive MI from week 1 to week 12, whereas DIG will receive the same MI intervention from weeks 13 to 24.Allocation concealment technique will be applied in process of randomisation. During weeks 1 to week 12, theIIG will perform self-directed physical activity and receive MI. On the other hand, the DIG will perform self-directed physical activity without MI. During week 13 to week 24, DIG will continue self-directed physical activity with MI. Whereas IIG will perform physical activity but will no longer be given MI.

## Methods of intervention

### Self-directed physical activity

Participants will perform a 24-week period of self-directed, pedometer controlled physical activity [38, 39]. All participants will be given a step-count tracker devise (Fitbit Alta HR) and investigators will explain the prescribed pedometer-based activity to participants. Fitbit Alta HR is a wearable gadget and can be used to monitor step count. The participants’ activity engagement will be accompanied by individual face-to-face and phone call MI [22]. Participants will be taught how to operate the Fitbit and will be taken on a 10 min walk to experience a moderate level of exertion.. At week 1 (baseline or T1), all participants will be prescribed to assess and record their activity using the Fitbit monitoring device through its application via computer or smartphone to establish their baseline physical activity volume by step count. Following this, they will be advised to perform their activity on their own pace as tolerated and will wear the Fitbit during the daytime or when they are available for physical activity throughout the 24 weeks. Individual participants will be advised and motivated to gradually increase their daily steps on physical activity at moderate intensity exertion or at their perception of taking some efforts but can talk during physical activity (the recommendation by the department of health, Australian government) [40] during face-to-face and over the phone MI. The participants will be advised for safety during physical activity. They will be suggested to stop physical activity if adverse symptoms occur such as chest pain or pain down to arms, dizziness, difficulty breathing, unusual rapid heart rate, and sever fatigue. The researcher will suggest to the participant to inform their family members and take a mobile phone with them before they go out to exercise. They will be also advised to take record of their daily steps in their notebook. All participants will be informed that the researcher will track the participants’ step count and tracker usage time via Fitbit connect application. Individual average daily step count, and activity level will be assessed at baseline (T1), week 12 (T2), and week 24 (T3).

### Motivational interviewing

MI will be used in encouraging participants through open-end questions, friendly and supportive communication and induction of behavioral changes [22, 24]. Each MI will be conducted through four phases of conversation comprising: 1) Engage, 2) Focus, 3) Evoke, and 4) Plan [22]. The study will use the dialog of MI guided by Mentha Counselling and will be conducted by a counsellor who experienced MI There will be a 20 min face-to-face in week 1 and three phone MI sessions (15 min) at weeks 2, 4 and 9 for IIG. For DIG this will take place at weeks 12, 13, 15 and 20.

### Blood collection and storage

Approximately 20 ml of blood will be collected via a venipuncture at T1, T2 and T3 for both groups. Whole blood will be collected into a tube containing anticoagulant and centrifuged immediately after collection or on the same day approximately 7 h later.

Isolation of peripheral blood mononuclear cells (PBMCs) by density gradient configuration using Ficoll-Paque will be used for immune cell functions. PBMCs will be stored in refrigerator not longer than 1 day and then use flow cytometry to assess the composition of the isolated PBMC populations.

### Method of data collection and outcome measurements

#### Primary outcomes

##### Psychological health and QoL

Levels of stress, depression and anxiety will be assessed using the DASS 21 [41]. The DASS21 has been validated for breast cancer survivors and has good reliability [42]. In addition, the FACT-B version 4 [43] will be used to measure QoL. This cancer specific questionnaire is well validated and has good reliability and measures physical, social/family, emotional and functional well-being aspects and breast cancer specific conditions [43, 44]. Participant will complete these instruments at baseline; week 1 (T1), week 12 (T2), and week 24 (T3).

##### Immune function

PBMC cells (white blood cells) will be isolated from blood and PBMC assessed for changes at the cellular level by flow cytometric analysis [45] at baseline; weeks 1 (T1), 12 (T2), and 24 (T3). Cell surface markers, CD40, CD80, CD83, CD86, MHC-I, MHC-II, CD14, CD16, CD206, CD209 will be assessed by flow cytometry technique [45] to determine the changes after the program. In addition, the ratio of type 1 and type 2 T helper (Th1/Th2) cytokines secreted by monocytes and T cells will be determined to understand any cellular changes following exercise activity.

#### Secondary outcomes

##### Average daily step count

Participants will record their own daily steps through the Fitbit Alta HRapplication, upload their step count on their computer or smartphone and send the data to the researcher by email or phone call weekly. They can also record their steps from the tracker in a provided notebook. Researchers can track an individual’s daily step count via Fitbit Alta HR application on a computer or smartphone. The average daily step count will be calculated at baseline at each of the 12 weeks of the intervention program.

##### Adherence in step tracker usage

The participants’ adherence to self-directed physical activity will be evaluated by their compliance by wearing the Fitbit Alta HR. The adherence rate of fitness tracker usage can be an indicator for exercise program feasibility in breast cancer patients [46]. The adherence will be defined as step count tracker wearing time with data capture (daily hours and the number of wearing days) [46]. The mean of daily hours and number of wearing days per week will be calculated at the end of week 12 and week 24 of self-directed physical activity period [31].

##### Exercise self-regulation

The participant’s self-regulation for exercise will be assessed using the behavioral regulations in exercise questionnaire version 2 (BREQ2) [33] on three occasions. This five level-Likert scale-questionnaire has 19 items assessing exercise self-regulation which consists of five categories: external, introjected, identified, intrinsic and un-motivated [33]. The psychometric properties of the BREQ2 is adequate to assess self-regulation for exercise in breast cancer survivors [47].

##### Exercise barrier and task self-efficacy

The nine item rating barrier self-efficacy scale will be used to assess the confidence of participants performing exercise when experienced some difficulties. Task self-efficacy will be assessed with a four items scale. This questionnaire was validated and tested for internal consistency in women with breast cancer during treatment period [34]. Efficacy beliefs will be assessed at the start and end of the trial (see study outcomes measurement in Table 1).

**Table 1:** Study outcomes measurement for both groups of participant

Outcome measurement	Method	Week 1 Baseline (T1)	Week 12 (T2)	Week 24 (T3)
**Primary outcomes**				
Psychology health	DASS-21	x	x	x
QoL	FACT-B version 4 questionnaire	x	x	x
Immune cell functions	Isolation of peripheral blood mononuclear cells	x	x	x
**Secondary outcomes**				
Daily step count/Average activity volume assessment	Step count tracker	x	x	x
Adherence in pedometer usage	Step count tracker wearing time with data capture (daily hours and the number of wearing days)		x	x
Exercise self-regulation;	BREQ2	x	x	x
Exercise barrier and task self-efficacy	Exercise barrier and task self-efficacy rating scale	x	x	x

### Statistical methods

#### Sample size estimation and justification

Based on a power of .8, alpha of .05 and large effect size in change in FACT-B (Cohen’s d = 2.23) from previous studies [48] we calculated that a minimum of 53 participants are required. Considering a 10–20% drop-out rate in exercise studies we decided to recruit a minimum of 64 participants (*n* = 32 in each of the two conditions).

#### Analysis of data

Baseline descriptive statistics (mean, standard deviation for continuous data and percentage for categorical data) will be used to describe the distribution of personal data and variables between two groups of participant (e.g., age, breast cancer health history, body compositions, and blood pressure). Independent T-test will be used to compare means for continuous data and the Pearson Chi square will be used for testing the difference in distribution of a categorical variable at baseline [49].

Linear-mixed models will be used to identify the relationship between physical activity volume by step counting and mental health (DASS-21), QoL (FACT-B), self-regulation (BREQ2) and self-efficacy at T1, T2 and T3; the relationship between activity volume by step counting and immune biomarker changes at T1,T2, and T3. The models will also be used to control for the effect of covariates or confounding factors and to manage for missing data.

#### Data security and confidentiality

The patient’s personal information and health history which are necessary for this research study will be protected for their privacy and confidentiality [50]. All data of participants will be recorded adequately and stored in a secure, password protected databank Agreements involving data ownership and storage will be done between Western health and Victoria University. This study has planned to hold clinical trials research data for 15 years or more based on circumstances [36]. It is possible to keep files of hard copy and electronic files in a research office at Sunshine Hospital. A locked filing cabinet and a computer for research data can only be accessed by agreed members of the research team. More specifically, the researchers will have a backup or reserved storage [36]. For destruction of the data, hard copy will be shredded by hospital and university office’s shredder. Digital information will be destroyed by deleting or overwriting the files. According to the protection of participants’ privacy, personal, health related data and clinical outcomes will be kept and reported in coded or reversibly anonymised technique. Re-identifiable information will be used for data management (name will be removed and replaced by a code which can be re-identified for relating the different data sets and data verification) [36].

## Discussion

Self-directed physical activity in this clinical trial is considered a feasible practice in breast cancer survivors. More specifically, the combination of self-monitoring (step count) and MI have been shown to be important strategies for long-term adherence to physical activity behaviour in people living with cancer. This trial, will examine the efficacy of these health behaviour change techniques in self-directed physical activity. This is an important issue because this is a potentially low cost intervention which could be applied and implemented widely if successful. This study is guided by the Self-Determination Theory [32] and Social Cognitive Theory [26]. We anticipate that those who adhere to the program will be more intrinsically motivated at the end of the trial in comparison to those who do not. We will also explore whether the initial motivational orientation predicts adherence to the physical activity program. In addition, we would anticipate that successful adherence to the physical activity program will be associated with higher levels self-efficacy believes and reduced number of barriers to be physically active.

The primary outcomes of this study focuses on both psychological and physiological changes which can translate positively for their breast cancer clinical outcomes. The study expects the enhancement of their QoL, psychological health and immune biomarkers in 12 to 24 weeks self-directed physical activity by pedometor application combining 12 weeks motivational interviewing.

## Data Availability

Study materials as well as datasets or analysed data during the current study are available from corresponding authors for all acceptable requests.

## Abbreviations

PAPHIO study: Physical activity adherence psychological health and immunological outcomes study
QoL: Quality of life
IIG: Immediate intervention group
DIG: Delayed intervention group
BREQ2: The behavioral regulations in exercise questionnaire version 2
DASS21: A 21-item depression, anxiety and stress scale,
FACT-B: the Functional Assessment of Cancer Therapy-Breast
ANOVA: analysis of variance
MI: Motivational interviewing
PBMC: Peripheral blood mononuclear cell
ES: Effect size
HREC: Human research ethics committee
CD: Cluster of differentiation
